# In vitro selection for adhesion of *Plasmodium falciparum*-infected erythrocytes to ABO antigens does not affect PfEMP1 and RIFIN expression

**DOI:** 10.1038/s41598-020-69666-9

**Published:** 2020-07-30

**Authors:** William van der Puije, Christian W. Wang, Srinidhi Sudharson, Casper Hempel, Rebecca W. Olsen, Nanna Dalgaard, Michael F. Ofori, Lars Hviid, Jørgen A. L. Kurtzhals, Trine Staalsoe

**Affiliations:** 1 Department of Immunology, Noguchi Memorial Institute for Medical Research, University of Ghana, Legon, Ghana; 2 Centre for Medical Parasitology, Department of Clinical Microbiology, Rigshospitalet, Ole Maaløes Vej, 7602, 2200 Copenhagen, Denmark; 3 Department of Infectious Diseases, Rigshospitalet, Copenhagen, Denmark; 4 Centre for Medical Parasitology, Department of Immunology and Microbiology, Faculty of Health and Medical Sciences, University of Copenhagen, Copenhagen, Denmark

**Keywords:** Preclinical research, Infection

## Abstract

*Plasmodium falciparum* causes the most severe form of malaria in humans. The adhesion of the infected erythrocytes (IEs) to endothelial receptors (sequestration) and to uninfected erythrocytes (rosetting) are considered major elements in the pathogenesis of the disease. Both sequestration and rosetting appear to involve particular members of several IE variant surface antigens (VSAs) as ligands, interacting with multiple vascular host receptors, including the ABO blood group antigens. In this study, we subjected genetically distinct *P. falciparum* parasites to in vitro selection for increased IE adhesion to ABO antigens in the absence of potentially confounding receptors. The selection resulted in IEs that adhered stronger to pure ABO antigens, to erythrocytes, and to various human cell lines than their unselected counterparts. However, selection did not result in marked qualitative changes in transcript levels of the genes encoding the best-described VSA families, PfEMP1 and RIFIN. Rather, overall transcription of both gene families tended to decline following selection. Furthermore, selection-induced increases in the adhesion to ABO occurred in the absence of marked changes in immune IgG recognition of IE surface antigens, generally assumed to target mainly VSAs. Our study sheds new light on our understanding of the processes and molecules involved in IE sequestration and rosetting.

## Introduction

Malaria remains a major cause of infectious disease mortality and morbidity in many parts of the World^1^. It is caused by haemo-protozoan parasites of the genus *Plasmodium*, which infect and multiply within erythrocytes. The formation of rosettes, which is the binding of multiple uninfected erythrocytes to a central infected erythrocyte (IE), was first reported in the simian malaria parasite *P. fragile*^2^, but it is also common among human malaria parasites^3–7^. In two of these, *P. falciparum* and *P. vivax*, rosetting has repeatedly been associated with severe disease^8–11^, although the functional significance of rosetting is not fully understood^12^.

Rosetting involves multiple parasite-encoded ligands and host erythrocyte receptors^13^. Particular members of the three major parasite multi-gene families *var* (*P. falciparum* only)^14–16^, *rif*^17,18^, and *stevor*^19^ have been implicated as ligands, while a range of glycoproteins, proteoglycans, and carbohydrate moieties on the erythrocyte surface have been proposed as host receptors. These latter molecules include the blood group determinants A and B^20^, CD36^21,22^, heparan sulphate^23^, complement receptor 1 (CR1)^14^, and glycophorin C^19,24^. Soluble host proteins such as IgM^25–27^ and α_2_-macroglobulin^28^ also appear critical for at least some forms of rosetting, adding further to the complexity of the rosetting phenotype.

Blood group O (lack of blood group antigens A and B) has repeatedly been associated with protection from severe *P. falciparum* malaria^29,30^. This suggests that IE adhesion to A or B antigen, on erythrocytes (rosetting) or on endothelium (sequestration), is a risk factor for development of severe malaria. Identification of the parasite ligand(s) mediating binding to these receptors is therefore of interest. To achieve that, we selected IEs for their ability to adhere to blood group A, B and O blood group oligosaccharides in vitro by repeated panning on these antigens immobilized to plastic via bovine serum albumin, and examined associated changes in transcription of parasite genes encoding putative adhesion ligands.

## Results

### *Plasmodium falciparum*-infected erythrocytes can be selected in vitro for binding to ABO blood group antigens

**Figure 1:**
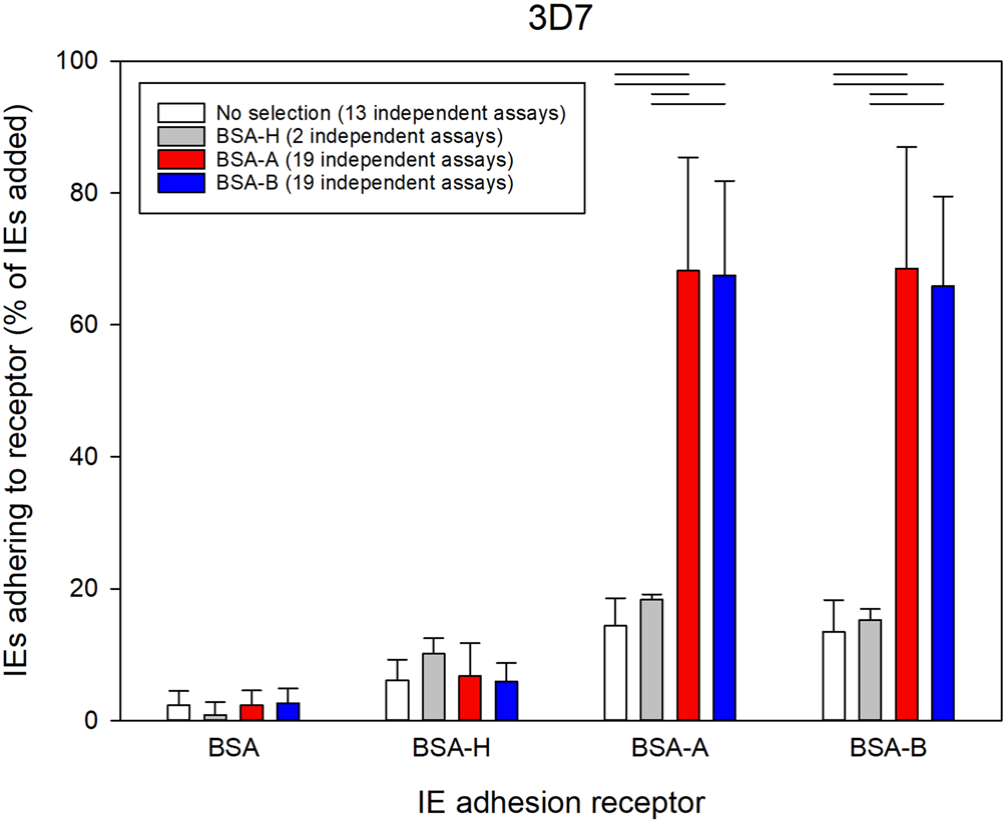
Selection for IE adhesion to ABO antigens. Adhesion of erythrocytes infected with late-stage *P. falciparum* 3D7 to BSA, BSA-H, BSA-A, and BSA-B before (white) and after four rounds of selection on BSA-H (grey), BSA-A (red), and BSA-B (blue), respectively. Adhesion of uninfected erythrocytes to the receptors was always < 5% of the erythrocytes added. Error bars indicate standard deviations of mean results from the number of independent assays indicated in the figure. Statistically significant differences (*P* < 0.05) of post-hoc pairwise comparisons (Holm-Sidak method) following 1-way ANOVA (*P* < 0.001) are indicated by lines along the top of the panel.

The blood group antigens A and B are formed by addition of a terminal α-1,3-*N*-acetylgalactosamine or galactose residue, respectively, to the H antigen (blood group O). To test the ability of IEs to adhere to these antigens in the absence of interference from other potential adhesion receptors present on erythrocytes and endothelial cells, we used bovine serum albumin (BSA) neo-glycoproteins displaying A, B, or H antigens (BSA-A, BSA-B, and BSA-H) as receptors for IE adhesion. Whereas unselected erythrocytes infected by *P. falciparum* 3D7 bound only weakly to any of these antigens, selection for IE adhesion to either BSA-A or BSA-B resulted in IEs that bound significantly better to both receptors, whereas selection for IE adhesion to BSA-H had little effect (Fig. 1). Selection of four additional *P. falciparum* lines/clones yielded results similar to those obtained with 3D7 for two of them (FMG and FUP), whereas we were unable to improve the adhesion to blood group sugars of erythrocytes infected by *P. falciparum* FCR3 or HB3 (Supplementary Fig. 1).

The similar results obtained by IE panning on BSA-A and BSA-B prompted us to verify the uniqueness of the neo-glycoproteins used. Blood group A-specific antibody blocked adhesion of IEs to BSA-A, but had only limited effect on adhesion of the same IEs to BSA-B (Supplementary Fig. 2). In contrast, blood-group B-specific antibody efficiently inhibited adhesion of IEs to BSA-B, but had no effect on adhesion of the same IEs to BSA-A (Supplementary Fig. 2). These results confirm that the BSA-A and BSA-B neo-glycoprotein constructs were antigenically distinct. To assess the possibility that the shorter (three-atom) spacer used in the BSA-H construct than in the BSA-A and BSA-B constructs (six-atom spacer) negatively affected IE adhesion to BSA-H, we also tested IE adhesion to a BSA-B construct with a three-atom spacer (BSA-B_short_). Although selected *P. falciparum* 3D7 bound less well to BSA-B_short_, similarly selected FMG IEs adhered equally well to either construct (Supplementary Fig. 3). This suggests that the length of the spacer is of limited importance.

**Figure 2:**
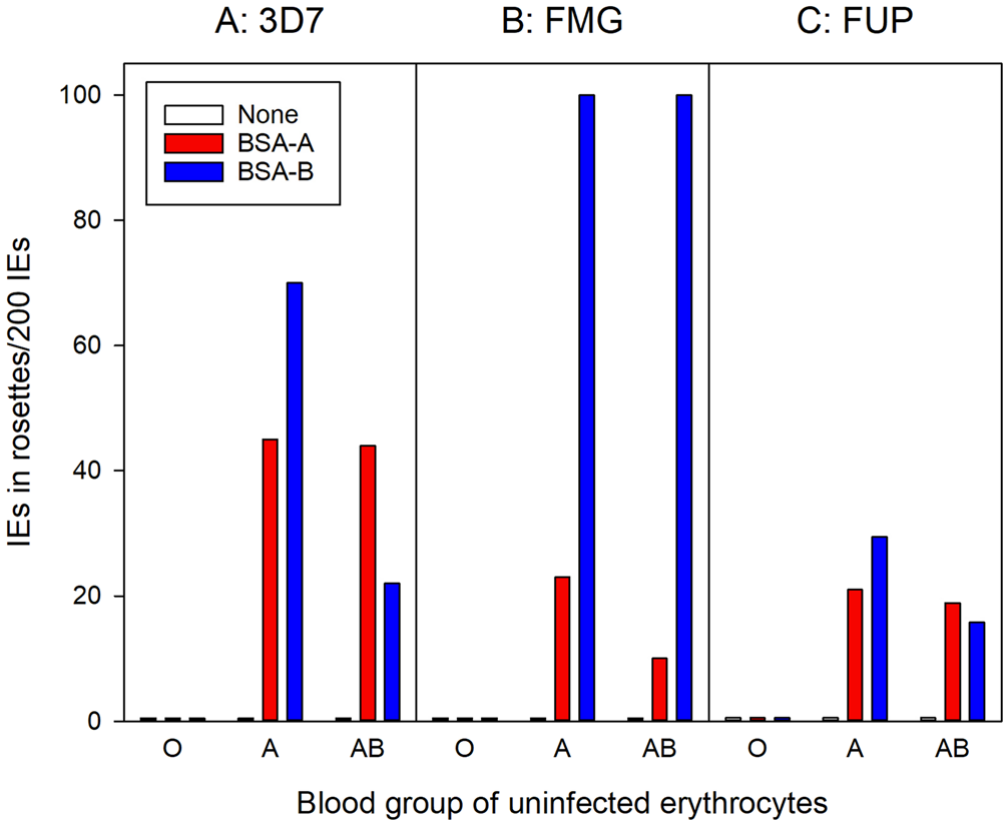
Formation of rosettes after selection for IE adhesion to ABO antigens. Frequency of rosettes formed by adhesion of uninfected blood group O, A, or AB erythrocytes to erythrocytes infected with late-stage *P. falciparum* 3D7 (**A**), FMG (**B**), or FUP (**C**) before selection (white) or after selection of IEs for adhesion to BSA-A (red) or BSA-B (blue). Data from one out of two independent experiments with similar results are shown.

**Figure 3:**
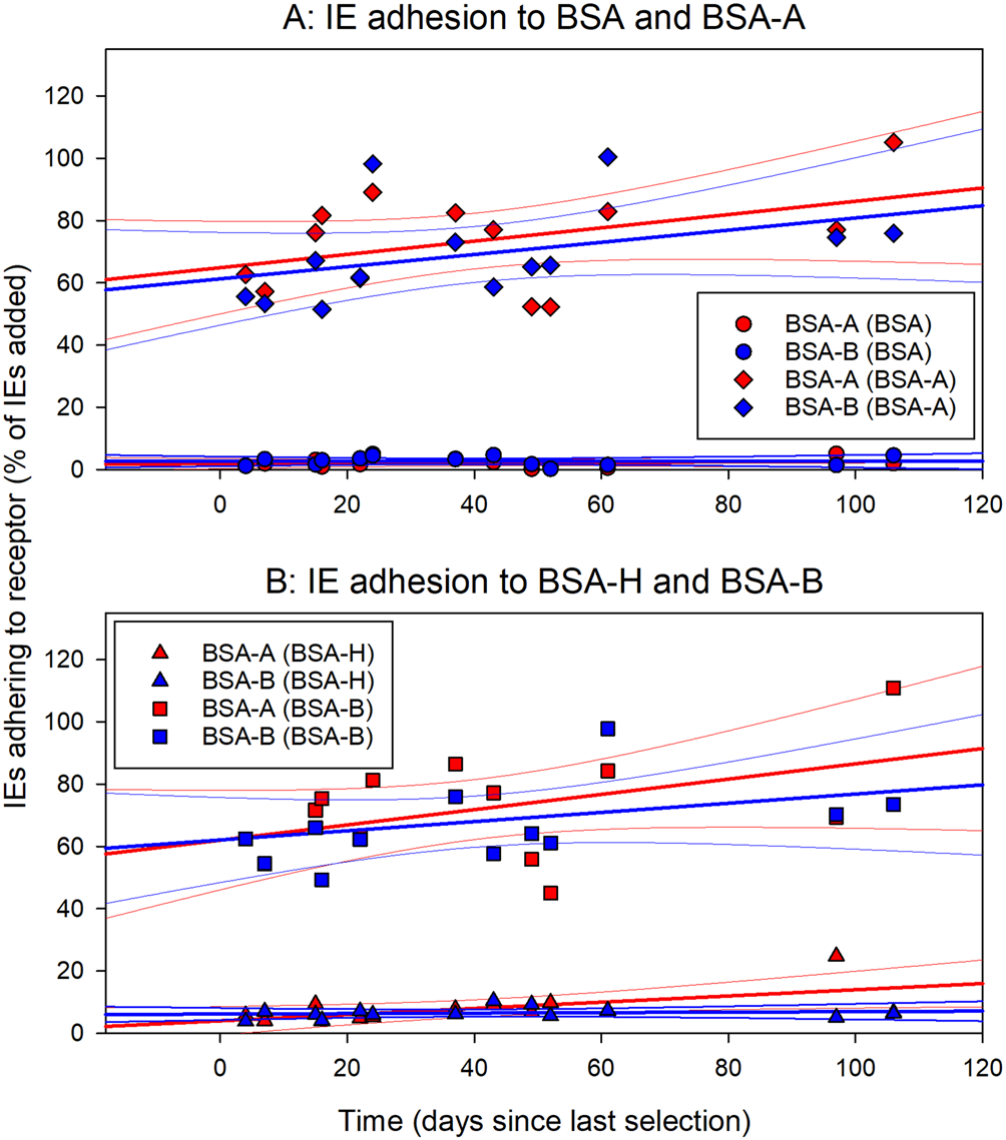
Temporal stability of IE adhesion phenotype following selection. Adhesion of *P. falciparum* 3D7-IEs to BSA (circles) or BSA-A (diamonds) (**A**), or to BSA-H (triangles) or BSA-B (squares) (**B**) at various time-points following selection for IE adhesion to BSA-A (red symbols) or BSA-B (blue symbols). Individual data points (symbols), regression lines (heavy lines), and 95% confidence intervals for regression lines (thin lines) are shown.

Overall, the experiments indicate that some, but possibly not all *P. falciparum* parasites can express ligands on the surface of IEs that enable their adhesion to the blood group carbohydrate antigens A and B.

### *Plasmodium falciparum*-IEs selected for adhesion to A and B blood group antigens form rosettes with corresponding uninfected erythrocytes

Several studies have implicated ABO antigens as receptors involved in the rosetting reaction around *P. falciparum* IEs. We therefore tested the impact on rosetting rates of selection for IE adhesion to ABO antigens. Without selection, rosettes did not form around erythrocytes infected by *P. falciparum* 3D7, FMG, or FUP, but selection of IEs for adhesion to either A or B yielded IEs that formed rosettes with A^+^ and AB^+^ erythrocytes, but not with O^+^ erythrocytes (Fig. 2 and Supplementary Table 1). These results support the importance of A and B blood group antigens in rosetting.

### Adhesiveness of *P. falciparum*-infected erythrocytes to blood group A and B antigens is a stable phenotype

The increase in IE adhesion to blood group A and B antigens following repeated panning of IEs on these receptors suggests that the adhesion is mediated by ligands encoded by members of one of the several multi-gene families in malaria parasites. While only limited information is available regarding the temporal stability of IE adhesion mediated by the STEVOR (~ 30 variants/parasite) and RIFIN antigens (~ 150 variants/parasite), substantial variation is known to exist in the stability of adhesion mediated by members of the best-studied adhesion family PfEMP1 (~ 60 variants/parasite). To determine the stability of the blood group A- and B-adhering IE phenotypes, in vitro cultures of *P. falciparum* 3D7 that had been selected for IE adhesion to either receptor were maintained for extended periods without further selection. In both cases, the initial IE adhesion phenotype was essentially stable for more than three months (> 50 generations) after the last selection round (Fig. 3).

### *Plasmodium falciparum*-infected erythrocytes selected for adhesion to A and B blood group antigens bind better to endothelial cells than unselected infected erythrocytes

**Figure 4:**
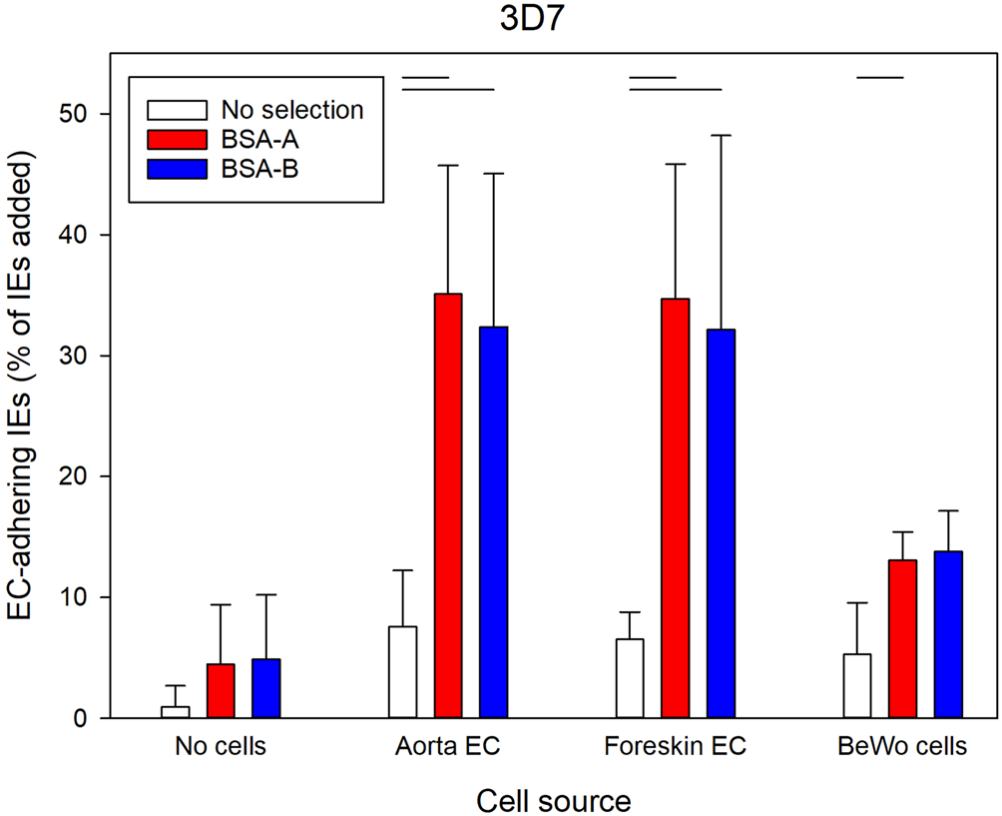
Adhesion of IEs to cellular receptors. Adhesion of erythrocytes infected with late-stage *P. falciparum* 3D7 to monolayers of aorta endothelial cells (EC), foreskin EC, BeWo choriocarcinoma cells, or uncoated wells (No cells) before (white) and after four rounds of selection on BSA-A (red) or BSA-B (blue), respectively. Error bars indicate standard deviations of mean results from four (No cells, Aorta EC, Foreskin EC) or three (BeWo cells) independent assays. Statistically significant differences (*P* < 0. 05) of post-hoc pairwise comparisons (Holm-Sidak method) following 1-way ANOVA (*P* < 0.01) are indicated by lines along the top of the panel.

Expression of ABO antigens is not restricted to erythrocytes, but is also widespread on vascular endothelium, where they are potential receptors for sequestering IEs. We therefore proceeded by comparing the ability of unselected IEs and IEs selected for adhesion to blood group A and B to adhere to cell lines and human primary endothelial cells. Erythrocytes infected by *P. falciparum* 3D7 (Fig. 4) or FMG (Supplementary Fig. 4A) and selected for adhesion to blood group A or B showed increased adhesion to primary human aortic and foreskin endothelial cells and to the choriocarcinoma cell line BeWo, compared to unselected IEs. In contrast, selected IEs did not bind better than unselected IEs to wild-type Chinese hamster ovary (CHO) cells (CHO-K1), to K1-derived glycosylation mutants A745 and D677, or to K1 cells transfected to express known *P. falciparum* adhesion receptors CD36 or CD54 (ICAM-1) (Supplementary Fig. 5).

**Figure 5:**
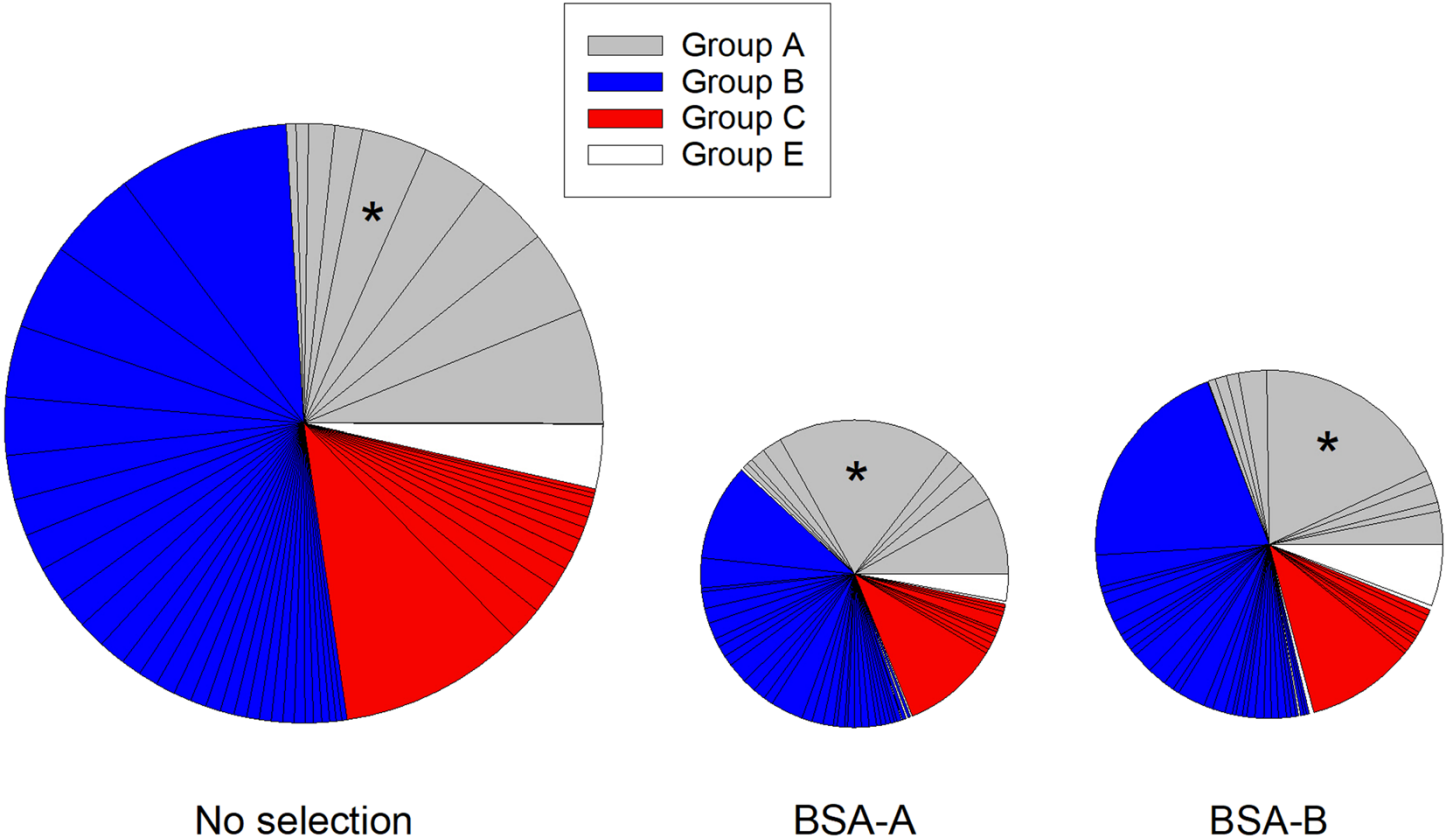
Transcription of *var* genes. Transcription of *var* genes in ring-stage *P. falciparum* 3D7 before (left) and after selection × 3 for IE adhesion to BSA-A (centre) or BSA-B (right). Genes are sorted according to structural groups ^79^ and to the transcript levels of individual genes relative to housekeeping gene in unselected parasites (this order is maintained in all panels). The size of the pies reflect the overall transcript levels relative to housekeeping genes. The *var* gene pf130003 is identified by an asterisk (*). For additional details regarding individual genes, see Supplementary Data.

*Plasmodium falciparum* HB3-IEs, which we were unable to select for increased IE adhesion to ABO antigens, adhered to the tested human endothelial cells to approximately the same degree as *P. falciparum* 3D7 and FMG after selection (Supplementary Fig. 4B).

### Selection of *P. falciparum*-infected erythrocytes for adhesion to A and B blood group antigens does not lead to major qualitative changes in *var* gene transcription

We next considered whether IE selection for adhesion to A and B antigens caused qualitative changes and/or quantitative increases in the transcription of genes encoding adhesive parasite ligands that could explain the increased IE adhesion to these receptors. As PfEMP1 appears to be the major IE adhesion ligands, we first compared levels of PfEMP1-encoding *var* gene transcripts in *P. falciparum* 3D7 and FMG before and after selection. Selection of IEs for increased adhesion to BSA-A and BSA-B tended to cause a reduction in the level of *var* gene transcripts relative to transcripts of housekeeping genes for both the tested parasites (Fig. 5, Supplementary Fig. 6). Although unselected and selected parasites alike were predominantly ring stages, as evidenced by microscopy and 10–30 × increases in transcript number of the ring stage-specific gene *sbp*-*1*^31^, fold increases tended to be lower for selected than for unselected parasites. Therefore, we cannot formally rule out that the apparent down-regulation was at least partially due to differences in the proportion of ring-stage parasites in the unselected versus the selected parasites. This notwithstanding, all data presented were obtained from a time point (15–16 h post-invasion), where transcription of both *var* and *rif* genes is active^32^.

**Figure 6:**
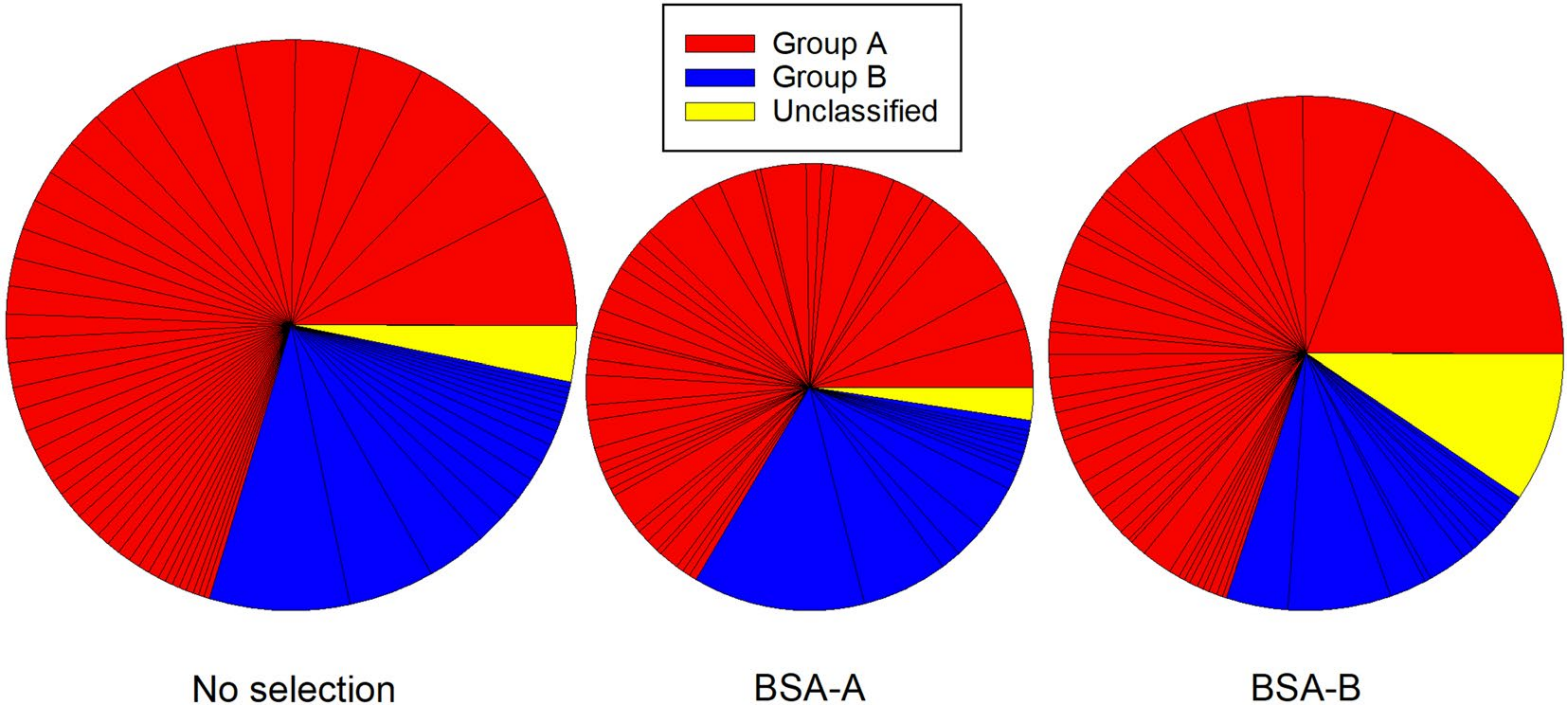
Transcription of *rif* genes. Transcription of *rif* genes in ring-stage *P. falciparum* 3D7 before (left) and after selection × 3 for IE adhesion to BSA-A (centre) or BSA-B (right). Genes are sorted according to types^77^ (**A**: red; **B**: blue; uncharacterized: yellow) and to the transcript levels of individual genes relative to housekeeping gene in unselected parasites. The size of the pies reflect the overall transcript levels relative to housekeeping genes. For details regarding individual genes, see Supplementary Data.

We observed no qualitative differences in the transcription of individual *var* genes (Supplementary Data), or in the relative proportions of transcribed genes in the structural *var* gene groups A, B, C, and E (Fig. 5 and Supplementary Fig. 6) that could explain the change in IE adhesion before and after selection. We were thus unable to substantiate the hypothesis that the observed increased IE adhesion to A and B blood group antigens in response to in vitro selection is due to changes in transcription of *var* genes encoding particular PfEMP1 adhesion ligands.

### Selection of *P. falciparum*-infected erythrocytes for adhesion to A and B blood group antigens does not lead to major qualitative changes in *rif* gene transcription

Particular RIFIN variants have also been implicated as parasite ligands involved in IE rosetting, and therefore by implication in endothelial IE adhesion^17,18,33^. We therefore proceeded to compare the levels of *rif* gene transcripts in *P. falciparum* 3D7 before and after selection. As for the *var* genes, selection tended to result in a reduction in *rif* gene transcript levels, in the absence of major qualitative differences at the level of individual genes or the proportion of genes in the structural *rif* gene groups A and B (Fig. 6, Supplementary Fig. 7). Increased IE adhesion to A and B blood group antigens in response to in vitro selection thus does not appear due to changes in *rif* transcription.

**Figure 7:**
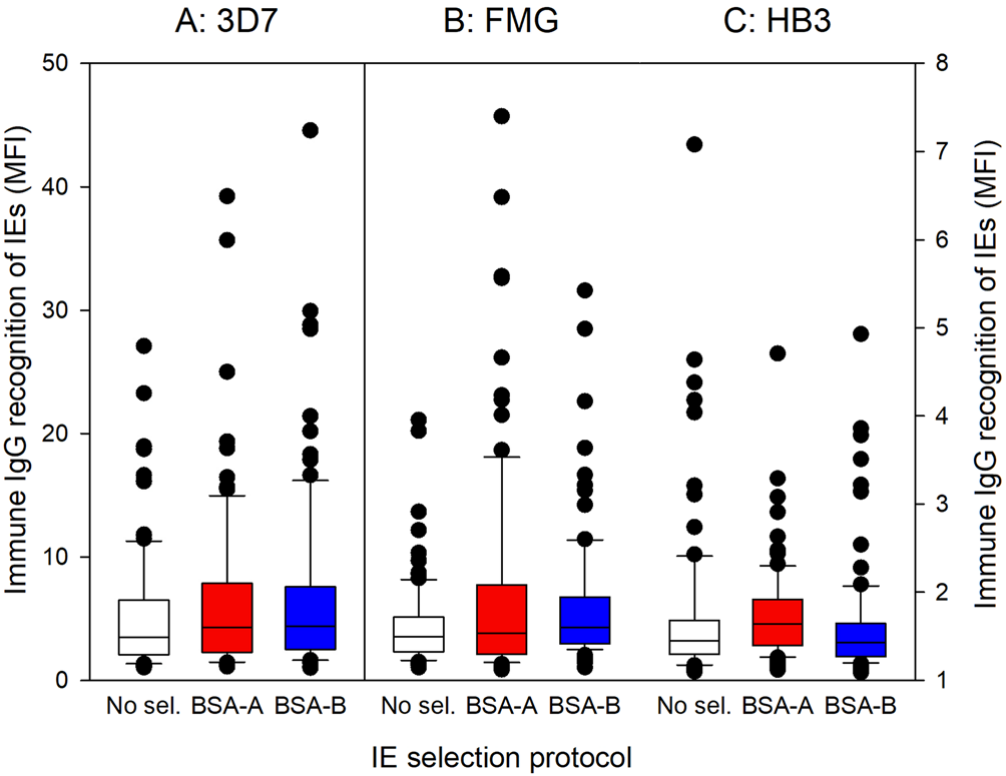
Immune IgG recognition of IEs. Immune IgG recognition of erythrocytes infected with late-stage *P. falciparum* 3D7 (**A**), FMG (**B**), or HB3 (**C**) before (white) and after four rounds of selection on BSA-A (red) or BSA-B (blue), respectively. Medians (centre lines), central 50% (boxes), central 90% (error bars), and outliers (circles) of levels of IgG in individual plasma samples from 93 Ghanaian children with *P. falciparum* malaria are shown. Recognition of the IEs by IgG in a pool of plasma from non-exposed donors was always < 5.5 (3D7) or < 1.5 (FMG and HB3).

### Selection of *P. falciparum*-infected erythrocytes for adhesion to A and B blood group antigens does not affect immune IgG recognition of the parasite ligands expressed on the IE surface

Rosetting has repeatedly been associated with severe *P. falciparum* malaria. Furthermore, protective immunity to the disease has been associated with acquisition of IgG specific for parasite antigens on the surface of IEs, and acquired protection against severe disease generally precedes protection against uncomplicated malaria and asymptomatic infection. On this basis, we hypothesized that selection for IE adhesion to ABO antigens would lead to increased recognition of the IEs by IgG from semi-immune individuals living in areas with stable transmission of *P. falciparum* parasites. To test this, we labelled unselected IEs and IEs selected for adhesion to blood group A and B antigens with plasma IgG from 93 Ghanaian children with *P. falciparum* malaria. For all the tested parasites (3D7, FMG, and HB3), the median IE recognition by immune plasma IgG was similar for unselected, BSA-A- and BSA-B-selected IEs (Fig. 7). Furthermore, immune IgG recognition of unselected and selected IEs were very significantly correlated for all three parasite lines (0.65 < r < 0.93; 1 × 10^–12^ < *P* < 1 × 10^–42^ for all comparisons). Recognition of different parasite lines selected the same way generally correlated much less well (0.08 < r < 0.64; 0.5 < *P* < 1 × 10^–12^ for all comparisons of parasites selected the same way). We thus found no evidence that selection for increased IE adhesion to blood group A or B antigens led to increased IE recognition by immune IgG, or to a different recognition pattern.

## Discussion

The majority of severe and fatal episodes of *P. falciparum* malaria occurs among young children in equatorial Africa. The resulting selection pressure on the human population has led to mutations and adaptations that confer some degree of clinical protection. Classical examples include sickle-cell anaemia and thalassaemia^34,35^. The ABO blood group antigens have also been implicated as determinants of malaria susceptibility, as blood group O appears to offer some degree of protection^36^. The association has mainly been attributed to the preferential involvement of the non-O blood group antigens in rosetting^20^. Rosetting can lead to vascular obstruction and endothelial inflammation, but likely also serves as a marker of adhesion of IEs to endothelial host receptors (sequestration), as blood group antigens are also widely expressed on vascular endothelium^37^.

Analysis of the role of ABO blood group antigens in malaria pathogenesis is complicated by the many other endothelial receptors known or suspected to be involved in sequestration, and by the high number and diversity of the parasite molecules that serve as sequestration ligands on IEs^38^. In the present study, we therefore sought to study the contribution of ABO antigens to IE adhesion and rosetting in the absence of other host receptors present on erythrocytes and endothelial cells. We also sought to gain insights regarding the parasite ligands mediating rosetting and sequestration.

To achieve this, we used five *P. falciparum* lines/clones (3D7, FCR3, FMG, FUP, and HB3) and commercially available BSA neo-glycoproteins, engineered to selectively display the H carbohydrate antigen (blood group O; BSA-H), blood group A antigen (BSA-A), or blood group B antigen (BSA-B). All the IEs used in our study initially showed minimal adhesion to each of the tested neo-glycoproteins (Fig. 1, and Supplementary Fig. 1). All except FCR3 and HB3 could be selected by repeated panning of IEs on BSA-A or BSA-B for markedly increased IE adhesion to these receptors. In contrast, attempts to select for increased adhesion to BSA-H (only 3D7) were unsuccessful. HB3 is genetically distinct from FCR3, whereas FCR3 is derived from FMG^39^, which responded rapidly to selection on BSA-A and BSA-B by changing its IE adhesion phenotype. FCR3 thus appears to have lost part(s) of its genome that is required for IE adhesion to ABO antigens. Alternatively, switching to that phenotype is for some reason a rare event in FCR3. Blood group O erythrocytes can form rosettes around IEs, but they tend to be smaller and weaker than rosettes involving A, B, or AB erythrocytes^20,40^. Several erythrocyte antigens have been implicated as receptors in rosetting^41^, and the most parsimonious explanation for our inability to select IEs for adhesion to the H antigen that defines blood group O is that the receptor mediating rosetting of blood group O erythrocytes is not the H antigen.

In our hands, selection on either BSA-A or BSA-B resulted in increased IE adhesion to both receptors (Fig. 1, and Supplementary Fig. 1) and in increased rosetting rates with both blood group A and blood group AB erythrocytes (Fig. 2). To our knowledge, selection for IE adhesion to ABO antigens in the absence of other potentially interfering receptors has not been reported before, but our results agree with reports implicating both blood group A and B antigens in rosetting^20^. The strong co-selection for IE adhesion to both A and B oligosaccharides by panning on either receptor indicates that the same parasite ligand(s) can bind both antigens, although this may not always be the case^42^. In addition to increased IE adhesion to BSA-A and BSA-B, selection on these receptors also lead to increased IE adhesion to several human cell lines (Fig. 4 and Supplementary Fig. 4). In contrast, the selection had little impact on IE adhesion to a range of CHO cells (Supplementary Fig. 5), including various glycosylation mutants and cells transfected to express human CD36 or ICAM-1, which are known *P. falciparum* IE adhesion receptors^22,43^. These findings support the hypothesis that the association between rosetting and severe malaria is not only mediated by rosetting per se, but also involves IE adhesion to endothelium^12^.

Proteins encoded by several parasite multi-gene families and expressed on the IE surface have been implicated in IE rosetting and endothelial adhesion^38^. We therefore sought to determine the role of these in the increased IE adhesiveness to BSA-A and BSA-B following selection. We first considered the PfEMP1 antigens, which are encoded by the approximately 60 *var* genes per genome. PfEMP1 is a key element in the characteristic clonal antigenic variation of *P. falciparum* that endows this species with the capacity for rapid changes in the antigenic and adhesive characteristics of IEs^44–46^. Furthermore, IE adhesion to a number of endothelial host receptors, including CD36, ICAM-1 (CD54), chondroitin sulphate A, and endothelial protein C receptor, has been attributed to particular PfEMP1 variants^47–50^. Severe disease is largely restricted to *P. falciparum* (where it has been associated with rosetting^8–10^, and although malaria-infected erythrocytes are generally able to form rosettes^2,4–7^, *P. falciparum* is the only malaria parasite infecting humans that possesses this type of antigens. Finally, PfEMP1 is believed to be the main target of naturally acquired protective antibodies targeting the IE surface^51,52^. Despite all these indicators, we did not find evidence of a marked change in *var* gene transcription profiles (Fig. 5 and Supplementary Fig. 6) that could explain the marked changes in IE adhesion to BSA-A and BSA-B (Fig. 1 and Supplementary Fig. 1) and in IE rosetting rates (Fig. 2) following selection for IE adhesion to blood group A and B. The only noteworthy difference observed was an increase in the proportion of *pf13_0003* transcripts following selection (Fig. 5). However, the change appears insufficient to explain the marked phenotypic response to selection, despite the fact that the PfEMP1 encoded by *pf13_0003* (and other genes with orthologous DBL1α domains, such as *varO* and *it4var9*) has previously been implicated in rosetting^14,53,54^. Indeed, transcription of *it4var9* appeared to be very low and did not change in response to selection (Supplementary Fig. 6 and Supplementary Data). The same was true for *it4var60*, another *var* gene implicated in rosetting through blood group A and other receptors^16^. Overall, IE selection for adhesion to blood groups A and B on *var* gene transcription tended to cause down-regulation relative to transcription of housekeeping genes (Fig. 5, Supplementary Fig. 6). The second major multi-gene family encoding antigens implicated in IE rosetting and adhesion to ABO antigens is *rif*^18,55^. However, the *rif* gene transcriptional response to IE selection for adhesion to ABO antigens was also minimal, apart from a similar tendency towards overall down-regulation (Fig. 6 and Supplementary Fig. 7). For both gene families, it cannot be formally excluded that this was related to a less stringent dominance of ring stages in the selected populations analysed.

The repertoire of *P. falciparum*-IE surface-reactive plasma IgG varies markedly with age, exposure, and level of clinical immunity to malaria^56–60^. Furthermore, in vitro selection of IEs for adhesion to specific host receptors can lead to dramatic changes in the IgG recognition of IEs—changes that correlate with the clinical immune status of the IgG donor^61,62^. These findings indicate that IE adhesion is mediated by parasite-encoded variant antigens on the IE surface, and has led to the identification of PfEMP1 and RIFIN proteins that are selectively expressed by IEs with defined adhesion phenotypes^18,48–50,63^. Despite this, we found here that IgG-specific recognition of IEs before and after selection for adhesion to ABO antigens were remarkably similar (Fig. 7).

There are several possible explanations for our findings. One is that IE adhesion to blood group sugars is not mediated by members of the variant surface antigen families PfEMP1 and RIFINs. Instead, it might involve variant antigens not studied here, e.g., STEVOR^19,64,65^, or unidentified conserved antigens. Our study has little to offer with respect to the former of these alternatives. With respect to the latter, our selection for IE adhesion to ABO antigens might conceivably have favoured parasites with reduced expression of PfEMP1 and/or RIFIN proteins, which would otherwise interfere with the functionality of the unknown parasite ligand. The absence of a serological response to the selection might reflect an increased IgG response to that ligand that offset a reduced IgG recognition of PfEMP1 and RIFIN antigens. However, the marked reduction in IgG reactivity with the IE surface if PfEMP1 expression is selectively disrupted^52^ can be seen as an argument against this hypothesis. Furthermore, the fact that erythrocytes infected by parasites recovered from severely ill patients tend both to be particularly well recognized by plasma IgG^59,60^ and to form rosettes^8,10^ is difficult to explain under this hypothesis.

Another possibility that potentially overcomes the above difficulties is that IE adhesion to ABO antigens depends on post-translational modifications of variant antigens. These modifications might enable increased IE adhesion to ABO antigens despite reduced VSA expression. Although this idea is currently unsupported by direct evidence, it is consistent with our findings. The remarkable—and somewhat surprising—temporal stability of the selection-induced change in IE adhesion to ABO antigens (Fig. 3) can also be accommodated in this scenario, if these putative modifications are assumed not to be restricted to particular members or types within the affected variant antigen families. Finally, the hypothesis is compatible with the bulk of the evidence available in the literature, and thus deserves further study.

In conclusion, we have shown that the study of IE adhesion to ABO blood antigens in the absence of other, potentially confounding host receptors can improve our understanding of the processes and molecules involved in IE sequestration and rosetting, two central elements in the pathogenesis of *P. falciparum* malaria.

## Methods

### *Plasmodium falciparum* malaria parasites

Five *P. falciparum* lines/clones were used in the experiments reported here. 3D7 is a clonal derivative of an isolate (NF54) originally obtained from a malaria patient in the Netherlands^66^. *P. falciparum* FMG was originally obtained from a Gambian patient, and is the parent of the widely used FCR3 line^39^. HB3 is a clonal derivative of an isolate from Honduras^67^, while FUP is a line originally obtained by inoculation of *Aotus* monkeys with blood from a patient returning from Uganda, and subsequently adapted to in vitro culture^68^. All the parasites were maintained in type O erythrocytes in RPMI-1640 medium supplemented with Albumax II, as described in detail elsewhere^69^. Late-stage (trophozoite/schizont) IEs were purified by magnet-activated cell sorting as described previously^70^, except that RPMI-1640 was used throughout. To rule out cross-contamination among the lines, or unintentional outgrowth of minor genotypes in non-clonal lines, we regularly verified the genotype of our cultures by monitoring line-specific polymorphisms in the MSP1 and MSP2 loci^71^.

### Cell lines

Primary human endothelial cells obtained from aorta and dermis of foreskin, and the choriocarcinoma cell line BeWo, were cultured in vitro according to recommended procedures as described elsewhere^72,73^. Chinese hamster ovary (CHO) cell lines A745, D677, K1 and K1-derived transgenic lines expressing human CD36 and CD54 (ICAM-1), respectively, were cultured in vitro as previously described^73^.

### ABO blood group oligosaccharides

Blood group A tri-saccharide with 6-atom spacer (NGP6305), blood group B tri-saccharide with 6-atom (NGP6323) or 3-atom (NGP0323) spacer, and blood group O (H) di-saccharide with 3-atom spacer (NGP0205), all coupled to bovine serum albumin (BSA), were obtained from Dextra, UK (https://dextrauk.com/). The ability of the constructs to bind and present the relevant oligo saccharide epitopes were confirmed with human ABO-specific, biotinylated plant lectins, and with A- and B-specific mouse monoclonal antibodies (Sigma SAB4700677 and SAB4700676, respectively) (Supplementary Fig. 8).

### In vitro selection of *P. falciparum* parasites for infected erythrocyte adhesion to ABO blood group antigens

ABO blood group saccharide-coupled BSA (10 µg/mL) was used to coat 24-well standard culture plates (400 µL/well, overnight, 4 °C). The wells were subsequently blocked with BSA (20 mg/mL, 2 h, room temp.), and washed × 3 in parasite culture medium. Purified late-stage IEs (~ 1 × 10^8^/mL) were added to the wells (400 µL/well) and incubated on a shaking table (60 min, room temp., 100 RPM). Unbound IEs were removed by gentle washing (×4 to ×6) with parasite culture medium (1 mL/wash) without initial removal of 400 µL. The wells were inspected using an inverted microscope after every other wash, to optimize enrichment of adherent IEs). After the final wash, parasite culture medium (500 µL/well) and uninfected erythrocytes (40 µL/well) were added to the plates, which were then incubated overnight under standard parasite culture conditions to allow reinvasion, followed by transfer to standard culture flasks and further propagation.

### Infected erythrocyte adhesion assays

The ability of IEs to adhere to ABO blood oligosaccharides and to cells was measured essentially as described previously^72^. Assays were done in 96-well Polysorb plates, coated (50 µL/well) and blocked (150 µL/well) as described for the selection procedure (BSA constructs), or in 96-well flat-bottomed tissue culture plates (cells), in which monolayers of the relevant cell type had been seeded approximately 48 h before. Wells with the appropriate cell culture medium but without cells were always included as negative controls. Purified late-stage IEs (1–2 × 10^7^/mL, 100 µL/well) were added to duplicate (adhesion to ABO constructs) or triplicate wells (adhesion to cells), and incubated (30 min on a shaking table as above). After the incubation, the plates were placed upside-down in a container with PBS (2% foetal calf serum; 60 min; room temp.) to allow unbound IEs to sink to the bottom of the container under gravity only. The plates were subsequently removed from the container, dried with paper tissue, followed by lysis of bound IEs by addition of PBS (100 µL/well), supplemented with Triton-X-100 (1% v/v). A separate preparation of lysed IEs (same number and batch as used in the adhesion assay) was then added to two previously empty wells (total_IEs). Adherent IEs (adh_IEs) were quantified by adding tetramethylbenzidine (100 µL/well) ELISA substrate and measuring the optical density (OD) of wells at 450 nm in a standard ELISA reader. The enzymatic reaction was stopped with sulphuric acid (1 M) while OD_total_IEs_ < 2 to ensure a linear relationship between the number of IEs OD. The fraction of IEs adhering was calculated as (OD_adh_IEs_ − OD_background_)/(OD_total_IEs_ − OD_background_), where OD_background_ was the mean OD obtained in wells, where IEs had not been added. In assays measuring IE binding to cells, the background wells were seeded with the appropriate cells.

The ability of anti-A and anti-B antibodies to inhibit IE adhesion to BSA-A and BSA-B was tested using saturating concentrations of the above-mentioned monoclonal antibodies.

### Rosetting assays

Purified late stage-IEs were mixed with uninfected erythrocytes with different ABO types. Incubation, staining with ethidium bromide and reading of the assays were as described previously^26^.

### Assessment of human IgG reactivity with variant surface antigens on infected erythrocytes

Purified late stage-IEs (1 × 10^5^/plasma sample) were first incubated with human immune plasma (5 µL; 30 min; room temp.), obtained from 93 ABO-typed (with commercially available agglutination kits (Fortress Diagnostics, UK)) children with acute *P. falciparum* malaria, living in an area of high and seasonal transmission of *P. falciparum* parasites^74^. Further processing and quantification of IgG binding to IEs by flow cytometry were essentially as described previously^75^, using FlowLogic software (https://www.inivai.com/).

### *var* and *rif* gene transcription profiling

Parasite cDNA was generated from DNA-free total RNA prepared from ring-stage IEs (approximately 15–16 h post-invasion) and using SuperScript II reverse transcriptase and random primers, according to the manufacturer’s instructions (https://thermofisher.com). Levels of *var* and *rif* gene transcripts were measured using cDNA, QuantiTec SYBR Green PCR Master Mix (Qiagen), and gene-specific primer pairs for 58 (3D7)^76^ and 56 (FMG)^71^ *var* genes, 154 *rif* genes (3D7)^77,78^, and for the endogenous control genes seryl-tRNA synthetase (p90) and fructose-bisphosphate aldolase (p61)^76^. Transcript copy number of each gene in tested cDNA was calculated using quantitative measurements of tenfold dilutions of genomic DNA as previously described^78^.

### Statistical analysis

The statistical significance of intergroup differences were first tested by 1-way analysis of variance. If that yielded a statistically significant (*P* < 0.05), statistically significant (*P* < 0.05) pairwise differences were identified by the Holm-Sidak method.

### Ethics statement

The work described here was conducted in accordance with all relevant guidelines and regulations, and all donors provided their informed consent to participate prior collection of blood samples. The experimental protocols (including collection of the human blood samples used), were approved by the Ethics Committee of the Noguchi Memorial Institute for Medical Research, University of Ghana, by Ghana Health Service (GHS-ERC 08/05/14), and by the Regional Research Ethics Committees for the Capital Region of Denmark (Protocol H-4-2013-083).

## Supplementary information

Supplementary file.

Supplementary Datasets.
