# Supplementary material for: In vitro selection for adhesion of Plasmodium falciparum-infected erythrocytes to ABO antigens does not affect PfEMP1 and RIFIN expression
Source: Sci Rep. 2020 Jul 30;10:12871. doi: 10.1038/s41598-020-69666-9 (PMC7393120; doi:10.1038/s41598-020-69666-9)
Supplement: Supplementary file 1 — Supplementary file. [file 41598_2020_69666_MOESM1_ESM.pdf]

SUPPLEMENTARY INFORMATION FOR

In vitro selection for adhesion of *Plasmodium falciparum*-infected erythrocytes to ABO antigens does not affect PfEMP1 and RIFIN expression

William van der Puije[1,2], Christian W Wang,[4] Srinidhi Sudharson,[2] Casper Hempel,[2] Rebecca W Olsen,[4] Nanna Dalgaard,[4] Michael F Ofori,[1] Lars Hviid[3,4], Jørgen AL Kurtzhals[2,4] and Trine Staalsoe[2,4#]

[1]Department of Immunology, Noguchi Memorial Institute for Medical Research, University of Ghana, Legon, Ghana. [2]Centre for Medical Parasitology at Department of Clinical Microbiology and at [3]Department of Infectious Diseases, Rigshospitalet, Copenhagen, Denmark. [4]Centre for Medical Parasitology at Department of Immunology and Microbiology, Faculty of Health and Medical Sciences, University of Copenhagen

Supplementary information

Supplementary Fig. 1. Selection for IE adhesion to ABO antigens

Adhesion of erythrocytes infected with late-stage *P. falciparum* FCR3 (**A**), FMG (**B**), FUP (**C**), or HB3 (**D**) to BSA, BSA-H, BSA-A, and BSA-B before (white) and after three (FCR3), four (FUP), five (FMG), or six (HB3) rounds of selection on BSA-A (red) or BSA-B (blue), respectively. Adhesion of uninfected erythrocytes to the receptors was always <5% of the erythrocytes added. Error bars indicate standard deviation of duplicate measurements.

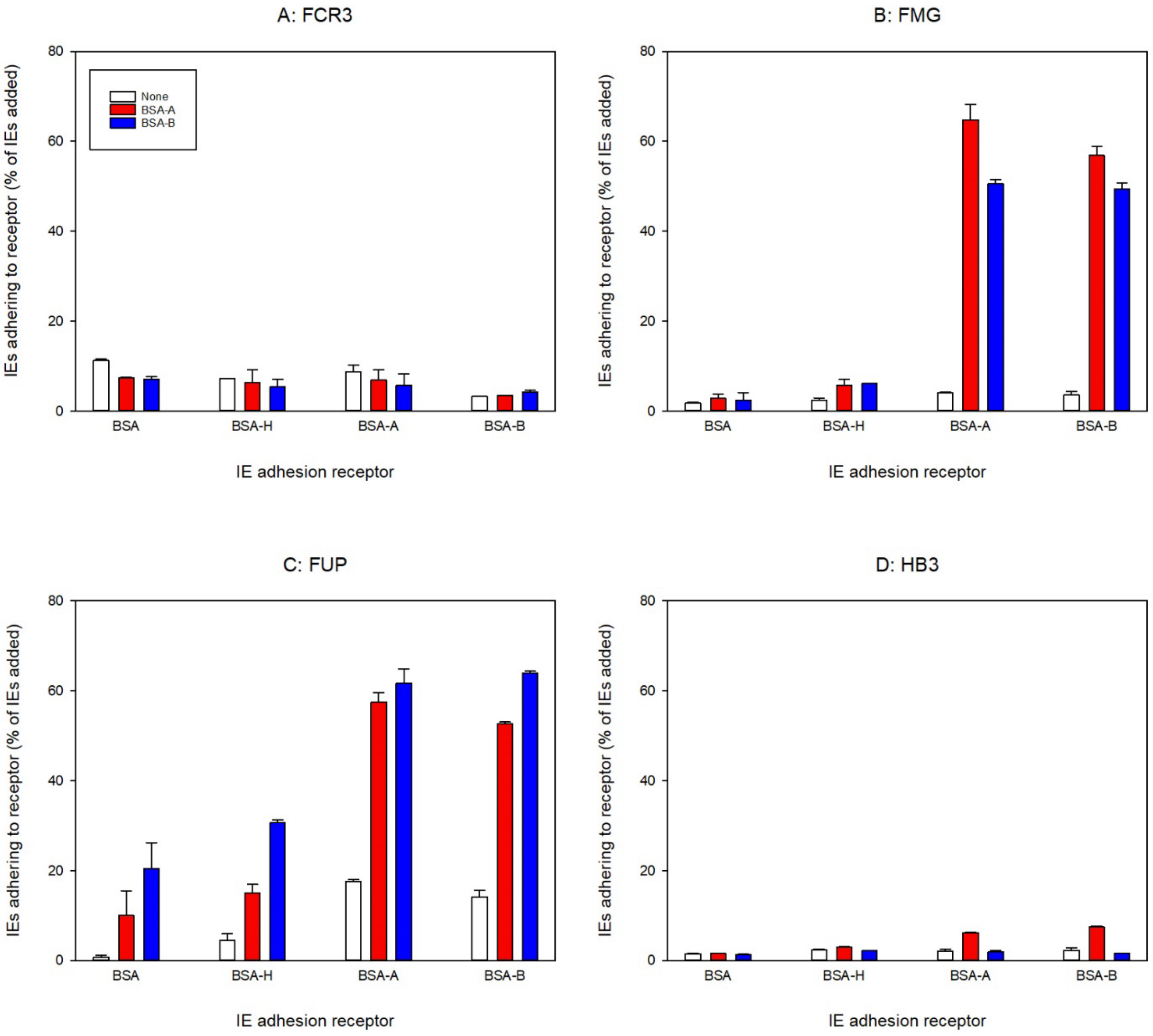

Supplementary Fig. 2. Inhibition of IE adhesion to ABO antigens by anti-Blood group A and anti-Blood group B antibodies

Adhesion of BSA-A-selected (A) and BSA-B-selected (B) *P. falciparum* 3D7 IEs to BSA-A (left) and BSA-B (right) in the absence of antibody (white) or in the presence of anti-Blood group A (grey) or anti-Blood group B monoclonal antibody (black). Error bars indicate standard deviation of duplicate measurements.

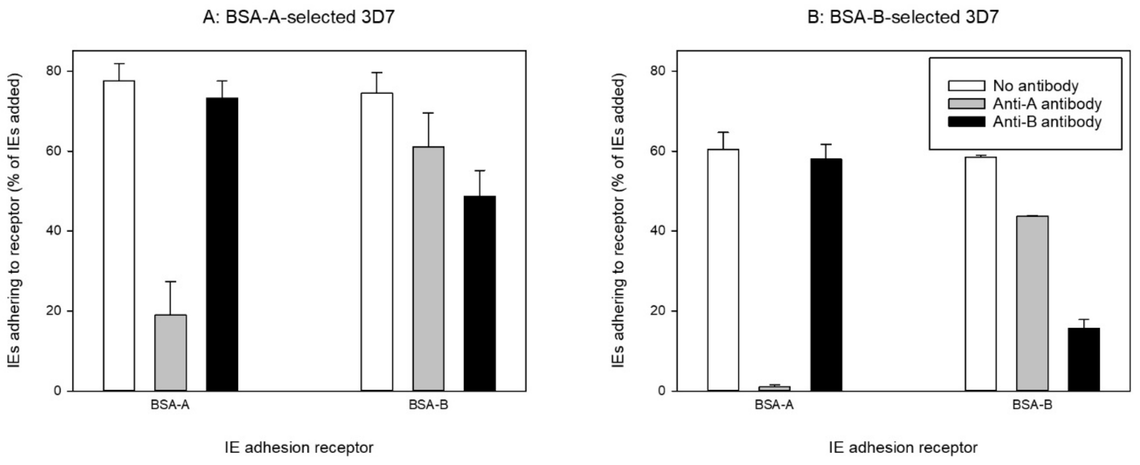

Supplementary Fig. 3. Adhesion of IEs to BSA-B and BSA-B$_{short}$

Adhesion of erythrocytes infected with BSA-A-selected late-stage *P. falciparum* 3D7 (**A**) or FMG (**B**) to BSA, BSA-H, BSA-A, BSA-B, and BSA-B$_{short}$. Adhesion of uninfected erythrocytes to the receptors was always <5% of the erythrocytes added. Error bars indicate standard deviation of duplicate measurements.

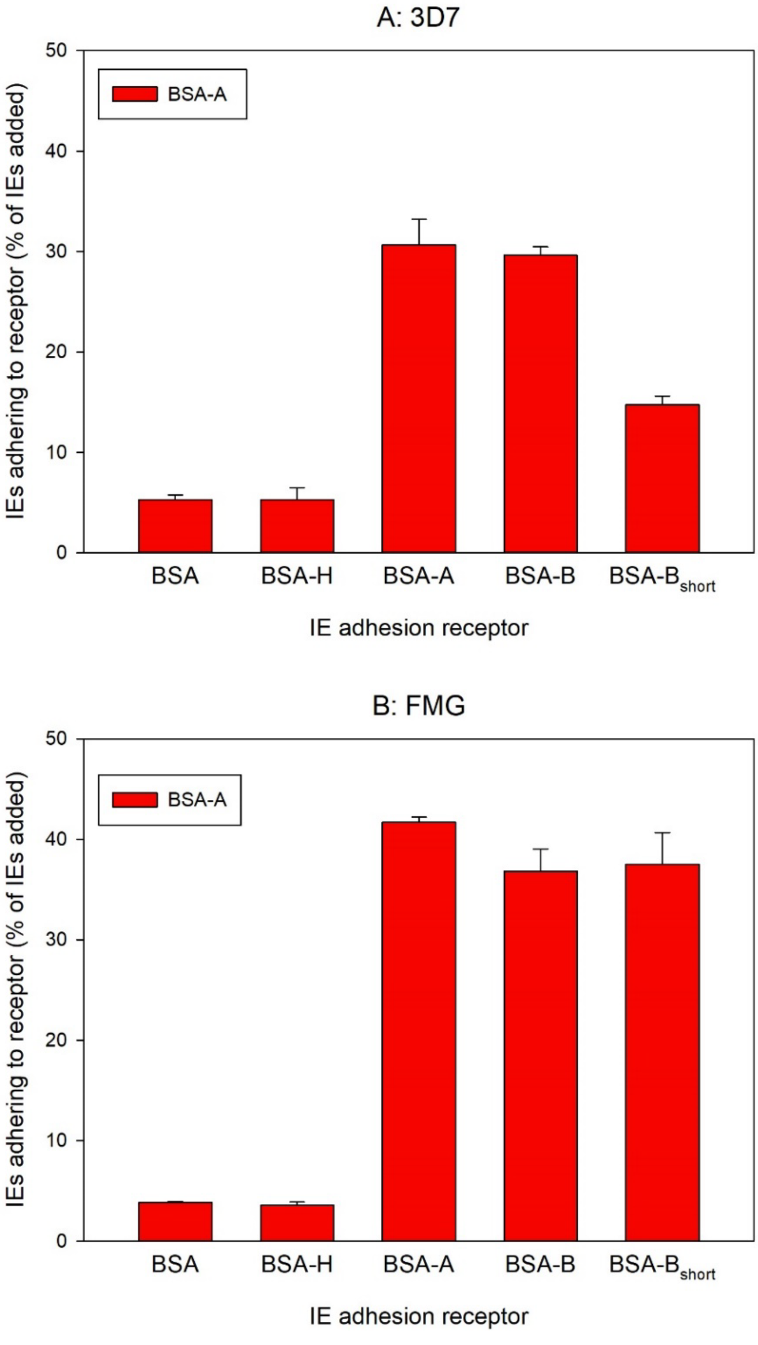

Supplementary Fig. 4. Adhesion of IEs to receptors on human endothelial cells

Adhesion of erythrocytes infected with late-stage *P. falciparum* FMG (**A**) or HB3 (**B**) to monolayers of aorta endothelial cells (EC), foreskin EC, BeWo choriocarcinoma cells, or uncoated wells (No cells) before (white) and after four rounds of selection on BSA-A (red) or BSA-B (blue), respectively. Error bars indicate standard deviation of triplicate measurements. Statistically significant differences (P<0.01) are indicated by lines along the top of the panel.

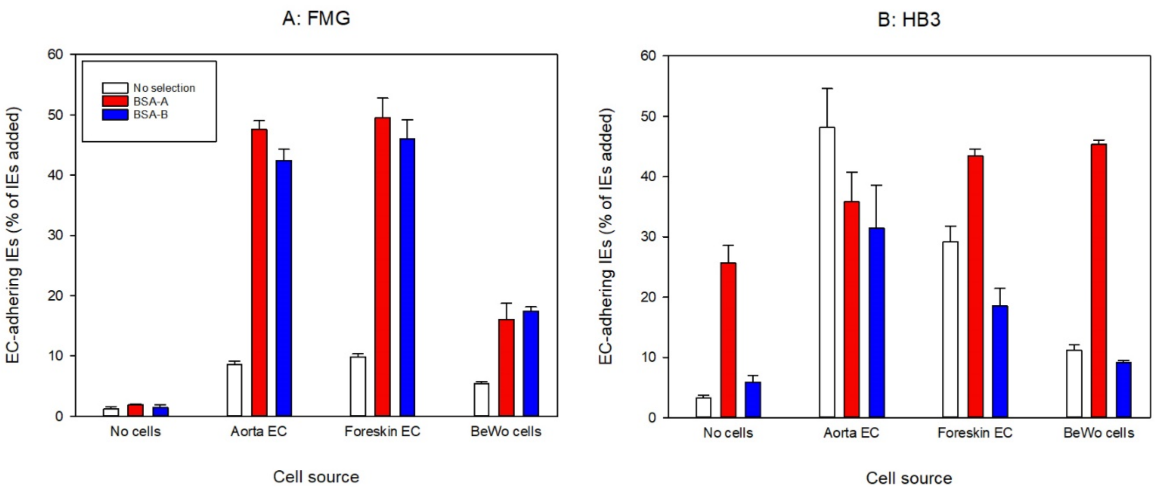

Supplementary Fig. 5. Adhesion of IEs to Chinese hamster ovary cell lines

Adhesion of erythrocytes infected with late-stage *P. falciparum* 3D7 (A), FMG (B), and HB3 (C) to monolayers of wild-type Chinese hamster ovary (CHO) cells (K1), CHO-K1 glycosylation mutants (K1-A745 and K1-D677), or to CHO-K1 cells transfected to express human CD36 (K1-CD36) or ICAM-1 (K1-CD54) before (white) and after >four rounds of selection on BSA-A (red) or BSA-B (blue), respectively. Error bars indicate standard deviation of triplicate measurements. Statistically significant differences (P<0.01) are indicated by lines along the top of the panel (no statistically significant differences observed).

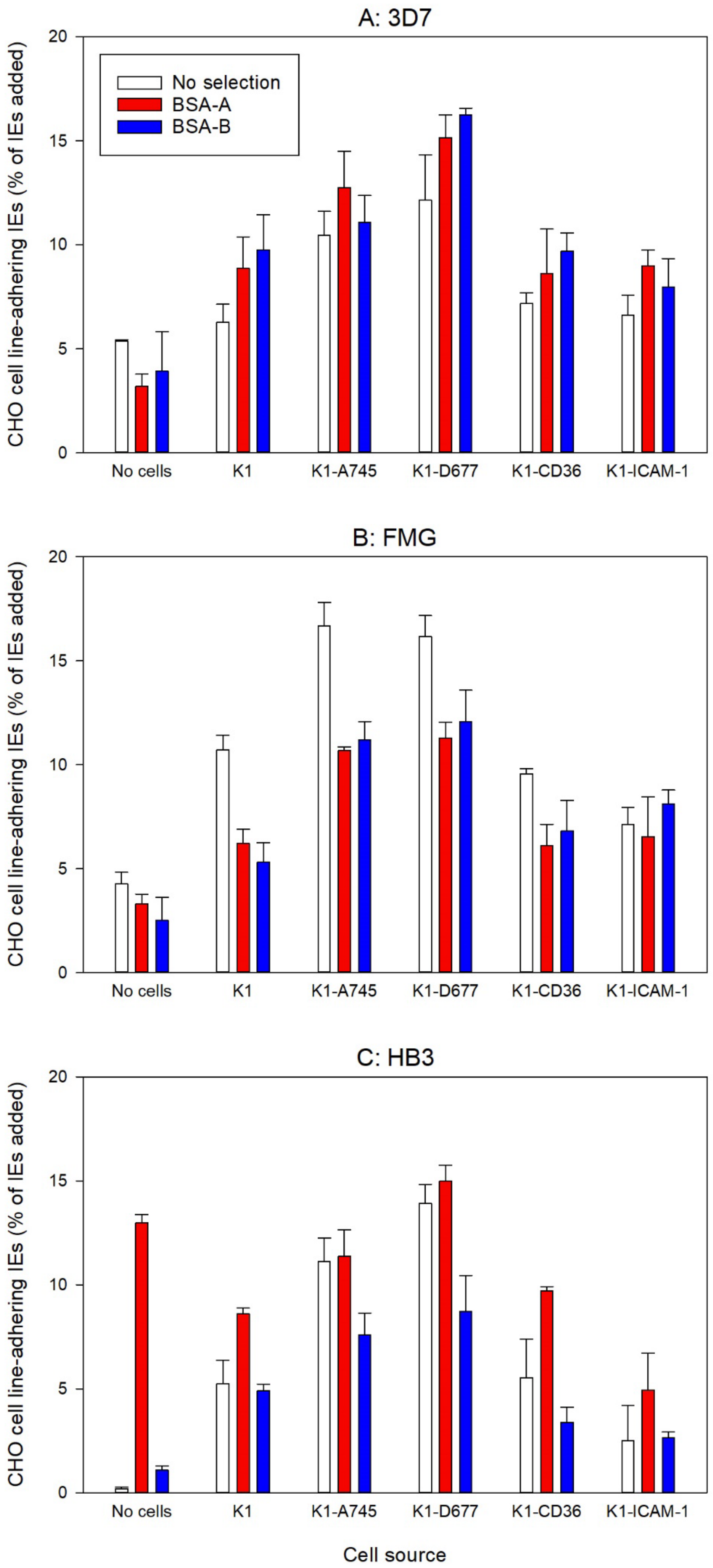
A: 3D7
B: FMG
C: HB3
No selection
BSA-A
BSA-B
CHO cell line-adhering IEs (% of IEs added)
0
5
10
15
20
No cells
K1
K1-A745
K1-D677
K1-CD36
K1-ICAM-1
Cell source

Supplementary Figure 6. Transcription of* var *genes (additional, independent experiments)

Transcription of *var* genes in ring-stage *P. falciparum* 3D7 (**A** and **B**) and FMG (**C**) before (left) and after selection for IE adhesion to BSA-A (centre) or BSA-B (right). The number of rounds of selection is indicated in each panel. Genes are sorted according to structural groups [1] and to the transcript level relative to housekeeping genes in unselected parasites. The size of the pies reflect the overall transcript levels relative to housekeeping genes.

A

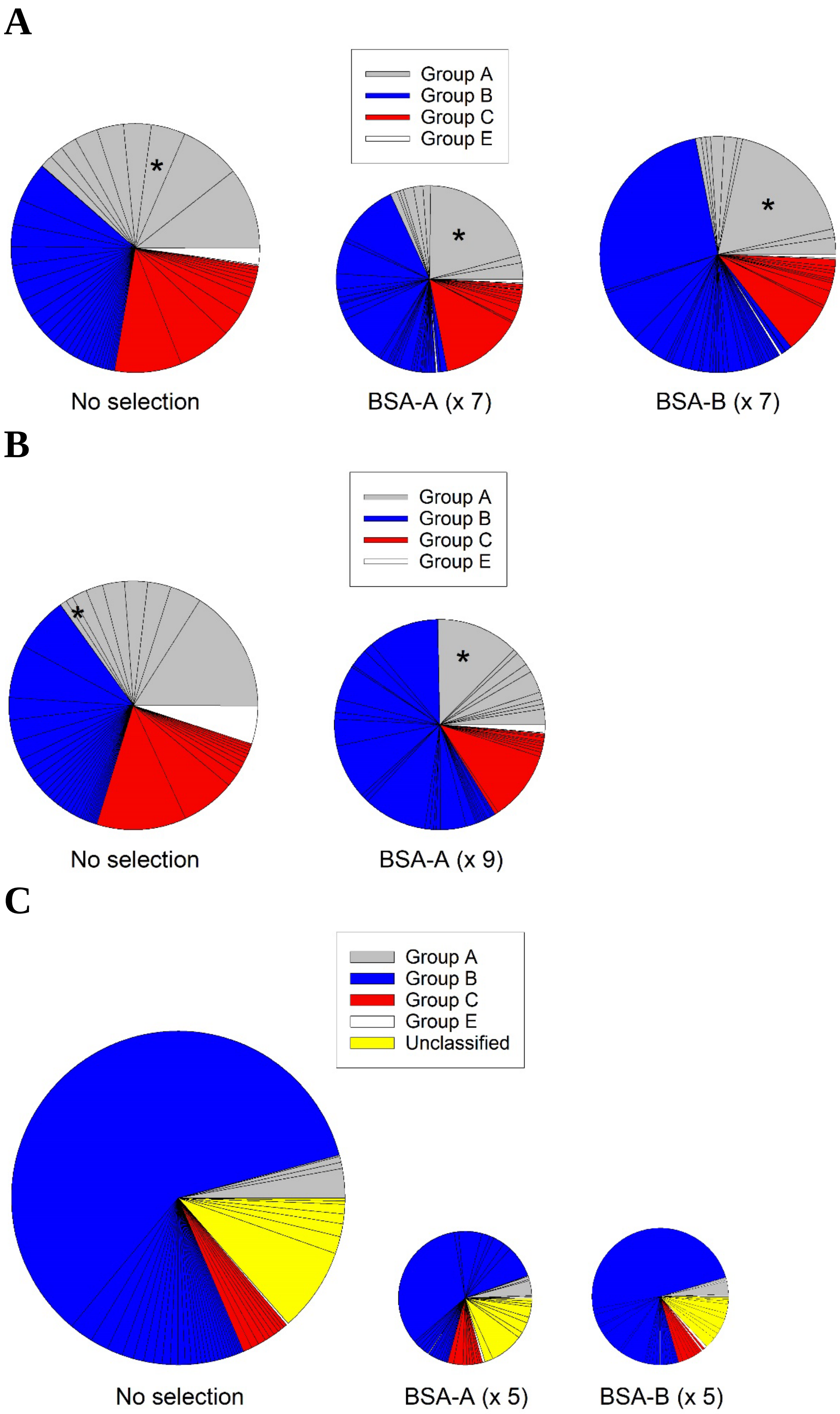
Group A
Group B
Group C
Group E
*
No selection
BSA-A (x 7)
BSA-B (x 7)
B
Group A
Group B
Group C
Group E
No selection
BSA-A (x 9)
C
Group A
Group B
Group C
Group E
Unclassified
No selection
BSA-A (x 5)
BSA-B (x 5)

Supplementary Figure 7. Transcription of* rif *genes (additional, independent experiments)

Transcription of *rif* genes in ring-stage *P. falciparum* 3D7 before (left) and after selection for IE adhesion to BSA-A (centre) or BSA-B (right). The number of rounds of selection is indicated in each panel. Genes are sorted according to structural groups [2] and to the transcript level relative to housekeeping genes in unselected parasites. The size of the pies reflect the overall transcript levels relative to housekeeping genes.

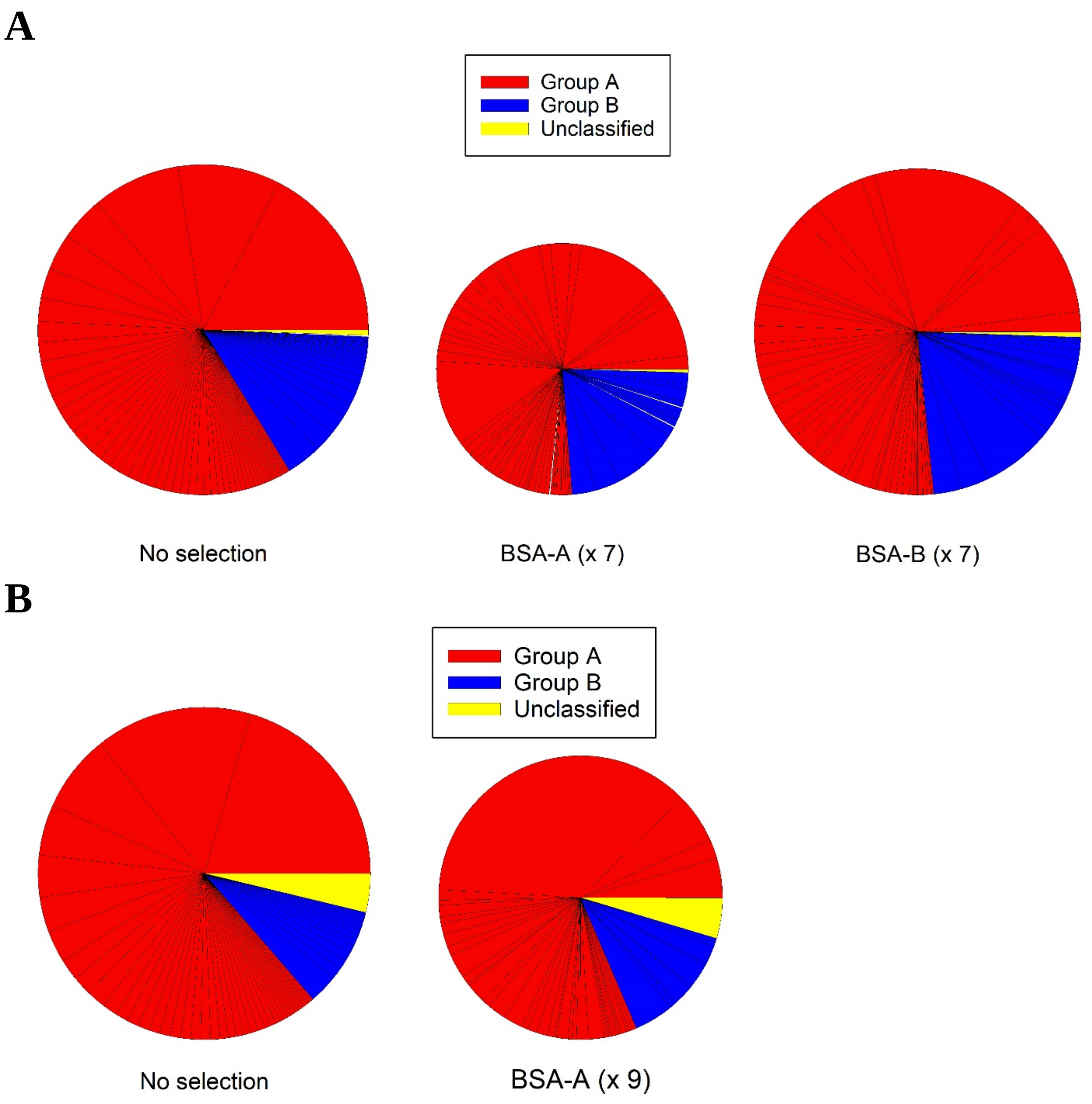

Supplementary Figure 8. Lectin and antibody typing of neo-glycoprotein reagents

Reactivity of BSA-H, BSA-A, and BSA-B neo-glycoproteins with *Ulex europaeus* agglutinin (UEA), *Galanthus nivalis* lectin (GNL), and *Dolichos biflorus* agglutinin (DBA) (left panel) and of BSA-A and BSA-B with mouse-anti-A and mouse anti-B monoclonal IgM (right panel).

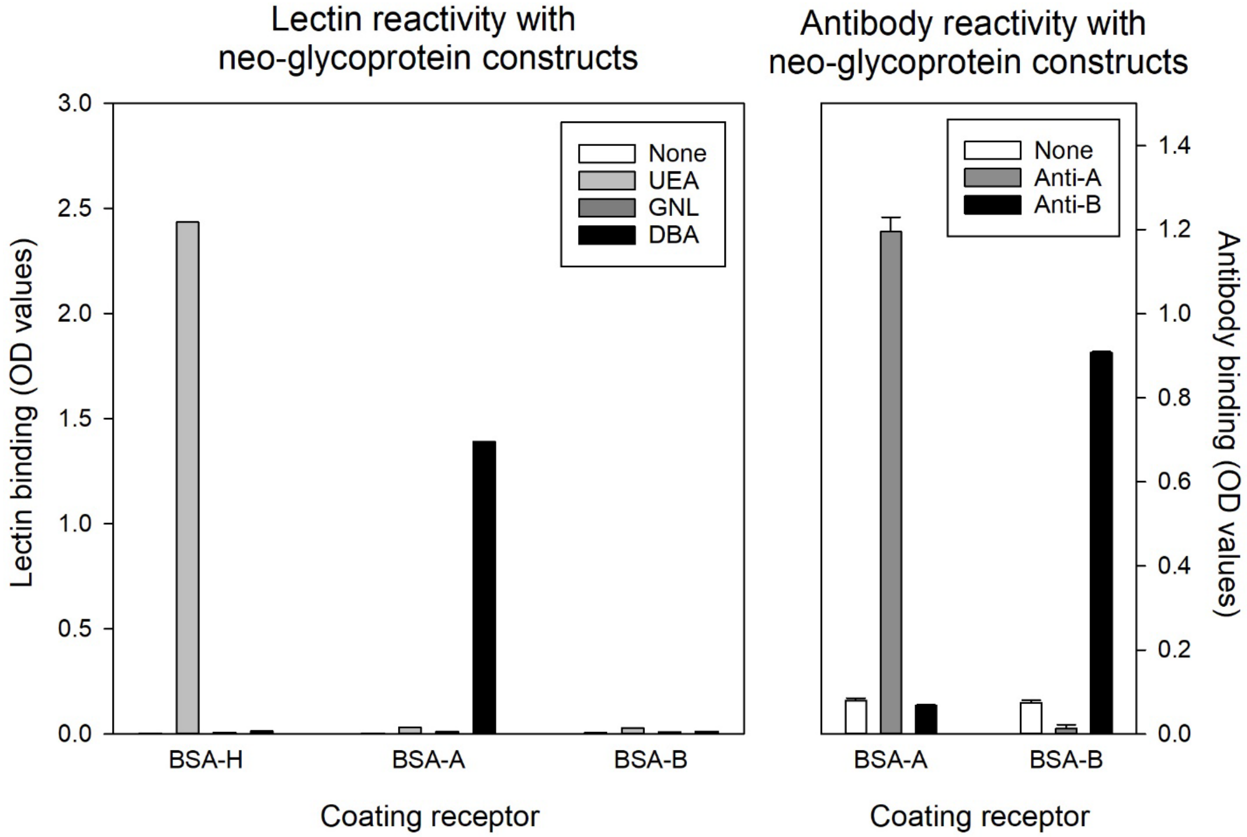

Supplementary Table 1. Percentage of IEs forming rosettes with erythrocytes

Clone	Selection	Blood group of uninfected erythrocytes			
		O	A	B	AB
3D7	None	0; 0*	1	2	0
	BSA-H	2; 0	3	0	10
	BSA-A	1; 0	95	80	94
	BSA-B	0; 0	41	69	67
FMG	None	0; 1 (3; 2)**	0 (2)	0 (4)	0 (6)
	BSA-H	n.d.	n.d.	n.d.	n.d.
	BSA-A	1; 0	42	14	54
	BSA-B	0; 0	43	15	42

n.d.: Not done. *Two donors tested. **Data in parentheses show percentage of IEs in auto-agglutinates (when present)

Reference

1 Rask, T. S., Hansen, D. A., Theander, T. G., Pedersen, A. G. & Lavstsen, T. *Plasmodium falciparum* erythrocyte membrane protein 1 diversity in seven genomes - divide and conquer. *PLoS Comput Biol* **6**, e1000933 (2010).

2 Joannin, N., Abhiman, S., Sonnhammer, E. L. & Wahlgren, M. Sub-grouping and sub-functionalization of the RIFIN multi-copy protein family. *BMC Genomics* **9**, 19 (2008).
